# Is the Adolescent Brain at Greater Vulnerability to the Effects of Cannabis? A Narrative Review of the Evidence

**DOI:** 10.3389/fpsyt.2020.00859

**Published:** 2020-08-26

**Authors:** Grace Blest-Hopley, Marco Colizzi, Vincent Giampietro, Sagnik Bhattacharyya

**Affiliations:** ^1^ Department of Psychosis Studies, Institute of Psychiatry, Psychology & Neuroscience, King’s College London, United Kingdom; ^2^ Section of Psychiatry, Department of Neurosciences, Biomedicine and Movement Sciences, University of Verona, Italy; ^3^ Department of Neuroimaging, Centre for Neuroimaging Sciences, Institute of Psychiatry, Psychology and Neuroscience, King’s College London, United Kingdom; ^4^ South London and Maudsley NHS Foundation Trust, London, United Kingdom

**Keywords:** cannabis, marijuana, adolescence, neurodevelopment, neurofuctioning

## Abstract

Cannabis use during the critical neurodevelopmental period of adolescence, may lead to brain structural, functional, and histological alterations that may underpin some of the longer-term behavioral and psychological harms associated with it. The endocannabinoid system performs a key regulatory and homeostatic role, that undergoes developmental changes during adolescence making it potentially more susceptible to the effects of exposure to cannabis during adolescence. Here, we synthesize evidence from human studies of adolescent cannabis users showing alterations in cognitive performance as well as in brain structure and function with relevant preclinical evidence to summarize the current state of knowledge. We also focus on the limited evidence that speaks to the hypothesis that cannabis use during adolescence, may pose a greater risk than use during adulthood, identify gaps in current evidence and suggest directions for new research. Existing literature is consistent with the association of cannabis use during adolescence and neurological changes. Adolescence cannabis users show altered functional connectivity within known functional circuits, that may underlie inefficient recruitment of brain regions, as largely increased functional activation has been observed compared to controls. This disruption in some cases may contribute to the development of adverse mental health conditions; increasing the chances or accelerating the onset, of their development. Preclinical evidence, further supports disruption from cannabis use being specific to the developmental period. Future studies are required to better investigate adolescent cannabis use with more accuracy using better defined groups or longitudinal studies and examine the permanency of these changes following caseation of use. Furthermore, research is required to identify heritable risk factors to cannabis use. There is a need for caution when considering the therapeutic potential of cannabis for adolescence and particularly in public discourse leading to potential trivialization of possible harm from cannabis use in adolescence. Current evidence indicates that adolescence is a sensitive period during which cannabis use may result in adverse neurocognitive effects that appear to show a level of permanency into adulthood.

## Background

Cannabis is the most used illicit drug worldwide, with those who go on to become habitual users most commonly beginning use during adolescence (1, 2). Attitudes are changing globally toward cannabis, with a trend toward legalization and a push for exploration into its medicinal potential. Therefore, consideration of possible harms associated with cannabis use, particularly during the vulnerable period of adolescence (3, 4), should be a key part of the discourse. Potentially greater vulnerability to harm from cannabis use during adolescence and their subsequent persistence has long been an area of scholarly focus, with numerous reviews aiming to tease apart the effects of cannabis use in adolescence compared to adulthood on cognition (5–9), neuro-structure (6, 10, 11), and neuro-functioning (6, 7, 10).

Adolescence is a critical period of neural development (12) and a later stage opportunity to sculpt the brain before a person reaches adulthood (13). Coupled with this, adolescence is also characterized by behavioral changes such as increased risk taking behavior (14) which may increase the likelihood of experimentation with drug taking (15). During infancy and childhood, brain growth focuses on increasing volume, producing more gray matter and neuronal synapses (16). During adolescence this shifts to creating more robust neuronal pathways. Useful neurons, dendrites, and synapses are selected for preservation, while others are pruned and eliminated, with increases in whole brain white matter occurring up until a person reaches their early twenties (17). During adolescent brain development, a decrease in grey matter can be observed in overall brain volume (18) as a result of pruning and eventual elimination of neurons in a ‘fine tuning’ of the brain, with cognitive maturation paralleling this elimination phase (13, 19, 20). Because of these reorganizational processes, the adolescent brain is highly sensitive to exogenous assault, such as from psychotropic substances, thereby posing a window of vulnerability to the emergence of developmental disturbances resulting from such exposure. This is particularly true of substances that target the endocannabinoid system, which, along with its other functions, plays a vital role in adolescent neuronal maturation (21).

The endocannabinoid system is a homeostatic regulatory system for various physiological processes (22, 23), playing a particularly important role during critical periods of developmental change (24, 25), through its retrograde inhibitory effect in an individual synapse-specific manner (26) acting *via* cannabinoid 1 (CB1) receptors, its main central receptor (27, 28). Exogenous assaults on this system, such as through cannabis use, may disrupt performance and cause desensitization or down regulation of receptors (29, 30). Adolescence represents a period of increased susceptibility to excitotoxicity from glutamate signaling (31, 32), which cannabis may further exacerbate through the mechanism of inhibiting GABAergic inhibitory action on glutaminergic neurons (33). Preclinical evidence suggests that during adolescence, the CB1 receptor shows a steadily increasing cortical expression, until stabilization in adulthood (34–36). An opposite pattern of expression is observed in the striatum, implying a role for CB1 receptor signaling in changes in the regulation of cortico-striatal transmission occurring during neuronal development (37, 38). A similar opposing pattern of changes are also seen with cortical and striatal dopamine synthesis levels, with levels going up in frontal regions and down in the nucleus accumbens and striatum during adolescence (39, 40). In light of the cross-talk between these signaling systems (41), dysregulation of both these neurotransmitter systems may result from exposure to exogenous cannabinoids during adolescence (42).

Patterns of cannabis use have been shown to be associated with greater harm in general when used regularly as opposed to infrequently; daily as opposed to once in a while; and when used in greater amount as opposed to smaller amounts (4, 43–46). Recent changes in cannabinoid consumption may have further increased the potential of harm in adolescent users. Potency of THC in herbal cannabis, the most common form of cannabis currently being consumed (47, 48), doubled in the 10 years up to 2005 in the UK (49) with similar patterns in Europe and the USA (50), and new preparations of cannabis such as resin oils may have up to a 75% THC content (51). Synthetic cannabinoids, which bind to the cannabinoid receptor 1 often with higher affinity making them potentially more harmful, are also being consumed increasingly nowadays (52, 53). Following legalization in some jurisdictions, commercialization of cannabis for use in vaporizing pens (54), drinks, sweets, and lollypops (55), may have made cannabis use more appealing to younger users and minimized the perceived harms of the drug (56).

A number of previous reviews have summarized evidence from animal and human studies focusing on the effects of cannabis use in adolescence (6–8, 57, 58). Comparing these alterations to those seen in adults (5, 9, 10), it has been suggested that adolescence may be a period of greater sensitivity to cannabinoid exposure. Here, we attempt to synthesize evidence from human studies of adolescent cannabis users showing alterations in cognitive performance as well as in brain structure and function with relevant preclinical evidence to summarize the current state of knowledge. We aim to also focus on studies that investigate the hypothesis that cannabis use during adolescence, a critical period of neurodevelopment, may pose a greater risk than use during adulthood, identify gaps in current evidence and suggest directions for new research.

## Methods

Human studies investigating cognition, brain structure and function following adolescent cannabis exposure, and animal studies directly comparing the cannabinoid exposure effects between adolescent and adult animals, were identified through a bibliography search of previous systematic and narrative reviews (5, 6, 10, 59, 60). To capture papers that have been published since the previous reviews, a search was carried out using the PUBMED database for relevant studies using the search terms “cannabis” or “marijuana” or “cannabinoid”, and “adolescence” or “young adult” or ‘early-onset”, which was completed on the 6/1/2020. These additional papers were screened initially through a search of titles and abstracts and finally a full article review. For the purposes of this review, we included studies that have specifically investigated alterations associated with adolescent cannabis use, compared early-onset cannabis users with later onset users, or used longitudinal design with a focus on effects of adolescent-onset use. Direct comparison of adolescent exposure with comparable extent of exposure during adulthood only in humans is the key piece of evidence that speaks to the central question addressed in this review (“Is the adolescent brain at greater vulnerability to the effects of cannabis as evident from structural, functional, and cognitive performance alterations?”). However, this is something that is difficult to directly address in human studies and has never been systematically investigated in humans to the best of our knowledge. To address this limitation in extant human evidence, we have summarized relevant preclinical evidence addressing this specific gap. We specifically focused on animal studies that directly compared cannabinoid exposure effects between adolescent and adult exposed groups as well as histological studies following adolescent cannabinoid exposure to address the gap in human evidence. Such controlled experimental evidence is lacking as far as human studies are concerned for obvious practical reasons. To further clarify, we have not systematically summarized the larger body of animal research related to the effects of cannabinoids, but only that relevant to our narrower focus on comparative studies using adolescent versus adult design.

## Results

Narrative synthesis of the different strands of evidence (human cognitive, structural imaging, and functional imaging evidence as well as preclinical evidence) relevant to the central issue under examination here are summarized under different sub-sections below and also presented in **Tables 1**–**4**, respectively.

**Table 1:** Studies investigating cognitive task performance in adolescent cannabis users.

Study	Task	CU (n)	NU (n)	Age of CU (years)	Age of NU (years)	Summary Results	Study design
Battisti et al. (61)	Verbal Memory Task	24	24	36.4 (11.2)	35.5 (11.5)	CU recalled significantly fewer words, which had a marginal correlation with the duration of use.	CU vs NU
Gruber et al (62)	Rey-Osterrieth Complex Gigure (visual memory) California Verbal Learning Test	34	28	22.76 (6.57)	24.32 (6.65)	No Significant difference seen between CU and NU. No significant difference seen between early or late onset CU.	CU vs NU Early (<16 years) Vs Late (>16 years) onset of cannabis use
Levar et al. (63)	California Verbal Learning Test	19	22	20.58 (2.52)	21.59 (1.94)	CU had worse performance, but only significant in the long-delay cued recall.	CU vs NU
Solowij et al. (64)	Rey Auditory Verbal Learning Test	52	62	18.67 (0.82)	18.07 (0.48)	CU recalled significantly fewer words than NU.	CU vs NU
Solowij et al. (65)	The Information Sampling Task	48	62	18.6 (0.8)	18.1 (0.5)	CU performed significantly worse than NU.	CU vs NU
Ehrenreich et al. (66)	Attention Test	Early: 48; Late 51		Early: 15.0 (1.1); Late 18.4 (1.8)		Early onset CU had impaired reaction time than Late CU.	Early (<16 years) vs Late (>16 years) onset of cannabis use
Fontes et al. (67)	Stroop Test Wisconsin Card Sorting Test Frontal Assessment Battery Wechsler Adult Intelligence Scale	Early: 49 Late: 55	44	Early: 30.4 (8.3); Late: 30.0 (4.1)	27.8 (8.0)	Early onset had significantly worse performance than Late and NU at attention, inhibition and executive function. No significant difference in vocabulary or IQ	Early (<15 years) of cannabis use vs Late (>15 years) of cannabis use vs NU
Pope et al. (68)	Battery of Ten Neuropsychological Tests	Early: 69 Late: 53	87	Early: 36 [32.5 -41]; Late: 44 [35.5–50]	40 [34–45]	Early onset CU performed worse on Verbal IQ then NU. Late had no significant difference in performance.	Early (<17 years) onset of cannabis use vs NU; Late (>17 years) onset of cannabis use vs NU
Castellanos-Ryan et al. (69)	Wechsler Memory Scales and Wechsler Adult Intelligence Scale	Cohort – 294				Age-of-onset for CU associated with decreased executive functioning and verbal IQ	Longitudinal study from age 10–20 years
Meier et al. (70)	Wechsler Adult Intelligence Scale-IV	Cohort – 1037				CU associated with reduced cognitive performance. Adolescent onset had increased IQ decline.	Longitudinal study at ages—18, 21, 26, 32 years

CU, Cannabis Users; NU, Non-Cannabis Using Healthy Controls.

**Table 2:** Studies investigating brain structural alterations using neuroimaging in adolescent cannabis users.

Study	CU (n)	NU (n)	Age of CU (years)	Age of NU (years)	Summary Results	Study Design
Arnone et al. (71)	11	11	25 (2.96)	23.36 (0.8)	CU increased mean diffusivity in the corpus callosum	CU vs NU
Ashtari et al. (72)	14	14	19.3 (0.8)	18.5 (1.4)	CU decreased FA in Bi. posterior internal capsule, L MTG, R STG	CU vs NU
Battistella et al. (73)	25	22	23 (2.2)	25 (2.8)	CU reduced GM in temporal pole and parahippocampal gyrus	CU vs NU
Bava et al. (74)	36	36	17.9 (0.9)	17.8 (0.8)	CU lower FA in L SLF, L postcentral gyrus, Bi. crus cerebri, R STG, R IFG. CU increased FA in Cuneus, R. SLF	CU vs NU
Cohen et al. (75)	19	17	21.5 (2.30)	22.7 (2.4)	Reduced GM associated with early onset cannabis use	CU vs NU
Epstein and Kumra (76)	19	29	16.6 (1.5)	16.5 (2.2)	CU had altered FA in the L inferior longitudinal fasciculus and L inferior fronto-occipital fasculus	CU vs NU
Gruber et al. (77)	25	18	23.16 (5.87)	23.11 (3.51)	CU decreased FA in Bi. genu of corpus callosum and L internal capsule	CU vs NU
Gilman et al. (78)	20	20	21.3 (1.9)	20.7 (1.9)	CU increased GM density in the L NA, subcallosal cortex, hypothalamus and amygdala	CU vs NU
Koenders et al. (79)	20	22	20.5 (2.1)	21.6 (2.45)	No significant difference	CU vs NU
Lopez-Larson et al. (80)	18	18	17.8 (1.0)	17.3 (0.8)	CU decreased middle frontal, superior frontal and insula cortical thickness	CU vs NU
Medina et al. (81)	16	16	18 (0.7)	18 (0.9)	No significant difference in WM or hippocampal volume	CU vs NU
Filbey et al. (82)	Early: 20 Late: 22		Early: 32.5 (8.01) Late: 30.25 (7.19)		No Significant difference in cortical thickness	Early (<16 years) Vs Late (>16 years) onset of cannabis use
Wilson et al. (83)	Early: 29 M-13, F-16 Late: 28 M-9, F-9		Early: M-31.5 (6.5), F- 33.2 (8.5) Late: M-27.9 (6.3), F-33.1 (6.7)		Early CU had increased WM volume	Early (<17 years) Vs Late (>17 years) onset of cannabis use

CU, cannabis users; NU, non-cannabis using healthy controls; M, male; F, female; R, right; L, left; Bi., bilateral; WM, white matter; GM, gray matter; STG, superior temporal gyrus; MFG, middle temporal gyrus; IFG, inferior frontal gyrus; SLF, superior longitudinal fasciculus; NA, nucleus accumbens; FA, functional anisotropy.

**Table 3:** Studies investigating brain functional alterations using functional magnetic resonance imaging in adolescent cannabis users.

Study	fMRI paradigm	CU (n)	NU 9n)	Age of CU (years)	Age of NU (years)	Summary Results	Study Design
Acheson et al. (84)	Win/lose gambling task	14	14	17.3 (1.3)	17.6 (1.0)	CU>NU MFG, caudate claustrum; CU>NU R MFG, R posterior &anterior cingulate, L insula, Bi. claustrum and declive.	CU vs NU
Behan et al. (85)	Go/no go task	17	18	16.5 (0.2)	16.1 (0.4)	NU>CU Bi. white matter adjacent to anterior cingulate.	CU vs NU
De Bellis et al. (86)	Decision-reward uncertainty task	15	23	16.4 (0.73)	15.4	CU>NU L SPL, L LOC< L. precuneus, R precuneus.	CU vs non-cannabis using controls with psychopathology
Jager et al. (87)	Monetary incentive delay task	21	24	17.2 (1.0)	16.8 (1.3)	No significant difference.	CU vs NU
Lopez-Larson et al. (88)	Finger Tapping	34	24	18.2 (0.7)	18.0 (1.9)	NU>CU R cingulate gyrus	CU vs NU
Schweinsburg et al. (89)	Spacial working memory task	15	17	18.1 (0.7) (SD)	17.9 (1.0) (SD)	CU>NU R SPL, NU>CU R dorsolateral PFC; CU>NU inferior cuneus.	CU vs NU
Schweinsburg et al. (90)	Verbal Encoding Task	36	38	18.1 (0.9) 18.0 (1.0)	17.6 (0.8) 18.1 (0.7)	No significant difference.	CU vs NU (also included binge drinking CU and NU groups)
Tapert et al. (91)	Go/NoGo Task	16	17	18.1 (0.7) (SD)	17.9 (1.0) (SD)	CU>NU Bi. SFG, Bi. MFG, R Insula, Bi. MFC, Bi. IPL, Bi. SPL, R lingual OG, R middle OG; CU>NU R IFG, R insula, R SFG, R MFG, R SPL, R IPL, R medial precuneus.	CU vs NU
Becker et al. (92)	Verbal Memory n-back task	Early: 26 Late:17		Early: 21.0 (2.8); Late: 24.5 (3.4)		No significant difference in cerebellum and DLPFC. Early > Late increased activation in the L.SPL	Early (<16 years) vs Late (>16 years) onset cannabis use
Gruber et al. (93)	Multi-Source Interference Task	Early: 9 Late: 14		Early: 21.44 (3.57); Late: 23.07 (6.20)		Early > Late onset increased activation in the mid R cingulum. Late > Early increased anterior L cingulum.	Early (<16 years) vs Late (>16 years) onset cannabis use
Sagar et al. (94)	Stroop Colour Word Test	Early: 24 Late: 26	34	Early: 23.67 (7.26); Late: 24.27 (6.79)	24.47 (6.49)	Early had activation pattern that included the L. anterior cingulate. Late had similar pattern to NU group.	Early (<16 years) vs Late (>16 years) onset cannabis users vs NU
Blanco-Hinojo et al. (95)	Resting-State Functional Connectivity	28	29	21 (2)	22 (3)	Abnormal FC between striatum and cortical area; striatum and ACC; striatum and fusiform gyrus.	CU vs NU
Orr et al. (96)	Resting-State Functional Connectivity	17	18	16.5 (0.2)	16.1 (0.4)	CU>NU Increased fALFF in SFG, RSPG, cerebellum. Decreased interhemispheric R SF, R SFG, pyramis of Cerebellum.	CU vs NU
Camchong et al. (97)	Resting-State Functional Connectivity	22	43	17.6 (2.4)	16.5 (2.7)	NU increased FC between ACC and SFG. CU decreased FC in caudal ACC and dorsolateral and orbitofrontal cortex.	Longitudinal design; CU vs NU
Thijssen et al. (98)	Resting-State Functional Connectivity	130	47	17.31 (1.09)	16.90 (1.19)	CU associated with decreased connectivity in precuneus network, auditory network, primary visual network. Increased connectivity between, R frontal-parietal and sensorimotor network.	Cohort of adolescence with (CU) and without cannabis use dependence (NU).

CU, cannabis users; NU, non-cannabis using healthy controls; M, male; F, female; R, right; L, left, Bi., bilateral; SFG, superior frontal gyrus; MFG, middle frontal gyrus; IFG, inferior frontal gyrus; MFC, medial frontal cortex; IPL, inferior parietal lobe; SPL, superior parietal lobule; OG, occipital gyrus; FC, functional connectivity.

**Table 4:** Preclinical studies comparing the effects of adolescent and adult cannabinoid exposure.

Study	Task/Testing	Adolescent Age of Exposure	Adult Age of Exposure	Washout Period	Animal Type	Drug Used	Results
Cha et al. (99)	Water Maze - Spacial task	34/36-55/57 PND	69/74-80/85 PND	28 days	Sprague Dawley rats	THC	No effect of drug was seen between groups
	Water Maze - Non-Spacial Task	34/36-55/57 PND	69/74-80.85 PND	28 days	Sprague Dawley rats	THC	No effect of drug was seen between groups
Cha et al. (100)	Water Maze	30-51PND	70-91PND	28 days	Sprague Dawley rats	THC	No effect of drug was seen between groups
Gleason et al. (101)	Prepulse inhibition	30-35 PND	63-70 PND	120 PND	Mice	WIN 55,212-2	Adolescence treated animals had deficits in inhibition compared to control animals. No deficit seen in adult treated animals compared to control animals.
	Fear Conditioning	30-35 PND	63-70 PND	120 PND	Mice	WIN 55,212-2	Adolescent animals had deficits in contextual learning compared to control animals. No deficits seen in adult treated animals compared to control animals.
Harte and Dow-Edwards (102)	Active Place Avoidance Testing	22-40 PND	41-60 PND	Adolescence: 33 days Adults: 16 days	Sprague Dawley rats	THC	Adolescent treated animals performed worse compared to control animals. No significant difference between adults and control animals.
Kasten et al. (103)	Object Recognition	35-44 PND	76-85 PND	Adolescent: 71 PND Adults: 117 PND	Mice	THC	No effect of drug was seen between groups
	CB1 receptor expression	35-44 PND	76-85 PND	Adolescent: 71 PND Adults: 117 PND	Mice	THC	Adolescent treated animals maintained increased CB1 receptor expression
O’Shea et al. (104)	Object Recognition	30-51 PND	56-77 PND	21 days	Wister rat	CP55,940	Adolescent treated animals had significantly poorer performance than adult treated animals
O’Shea et al. (105)	Object Recognition	30 -51 PND	56 -77 PND	28 days	Wister rat	CP55,940	No significant difference in either group
	Social Interaction Test	30 -51 PND	56 -77 PND	28 days	Wister rat	CP55,940	Adolescent treated animals had increased anxiety. No significant difference in adult treated animals.
Quinn et al. (106)	Object Recognition	28 PND	60 PND	10 – 15 days	Wister rat	THC	Adolescent treated animals had significantly impaired object recognition.
Renard et al. (107)	Object Recognition	29-50 PND	70-91 PND	28 days	Wister rat	CP55,940	Drug treated animals spent less time exploring novel objects and had significantly different times exploring familiar objects to control. No significant difference seen between adult animals and control group.
		29-50 PND	70-91PND	28 days	Lister Hooded rat	CP55,940	Drug treated animals spent less time exploring novel objects and had significantly different times exploring familiar objects to control. No significant difference seen between adult animals and control group.
	Object location	29-50 PND	70-91PND	28 days	Wister rat	CP55,940	Drug treated animals did not show a significant change to novel exploration time, where control did. No significant difference seen between adult animals and control group.
		29-50 PND	70-91PND	28 days	Lister Hooded rat	CP55,940	Drug treated animals did not show a significant change to novel exploration time, where control did. No significant difference seen between adult animals and control group.
Schneider and Koch (108)	Object Recognition	40-65 PND	> 70 PND	20-25 days	Wister rat	WIN55,212-2	Drug treated animals showed significantly impairment of recognition memory. No significant difference between adults and controls.
							Novel object recognition was significantly lower in drug treated animals to controls, however delay time had no significant effect between groups. No significant effect between adults and controls.
Dalton and Zavitsanou (109)	Acute CP55,940 Treatment	35 PND		24 hrs	Wister rat	HU210	Adolescent treated animals had a reduction in compensatory downregulation of CB1 receptors
Higuera-Matas et al. (110)	Hippocampal Microdialysis Testing	28 – 38 PND		93 – 96 PND	Wister rat	CP 55,940	Drug treated animals had increased GABA release in the hippocampus and increased GABA receptor expression
Renard et al. (111)	Electrophysiological analyses	35 PND		30 days	Sprague-Dawley rat	THC	Drug treated animals had decreased GABAergic levels in PFC compared to controls animals
Rubino et al. (112)	Hippocampal protein expression and binding analysis	35-45 PND		30 days	Sprague Dawley rats	THC	Drug treated animals had reduced VAMP2 expression and NMD receptor binging in the hippocampus
Rubino et al. (113)	Histochemical analysis	35 – 45 PND		1, 15 or 30 days	Sprague-Dawley rat	THC	Drug treated animals had decreased expression and sensitivity of CB1 Receptors and increased levels of NMDA receptors
Verdurand et al. (114)	[^35^S]*t*-butylbicyclophosphorothionate autoradiography	5 weeks	10 weeks	24 hours	Wister rat	HU210	Cannabinoid exposed rats had increased hippocampal binding. No significant effects of GABA receptors
Zamberletti et al. (115)	Histochemical analysis	35-45 PND		30 days	Sprague-Dawley rats	THC	Reduced levels of GABA in the PFC

CB1 receptor, cannabinoid receptor type 1; THC, delta-9-Tetrahydrocannabinol; GABA, gamma-aminobutyric acid; PFC, pre-frontal cortex; NMDA, N-methyl-d-aspartate; VAMP2, vesicle-associated membrane protein 2.

### Studies Investigating Alteration in Cognitive Task Performance in Adolescent Cannabis Users

Human studies (please also see **Table 1** for a list of studies and summary results) have reported impaired cognition across a number of domains (executive functioning, processing speed, attention, and memory) in adolescent cannabis users when compared to controls of the same age (8, 116, 117), with some suggestion that the magnitude of this impairment may be greater than in adult cannabis users particularly in the domains of learning and memory (89). Cognitive performance deficits spanning a range of executive function domains have been found in cannabis users to be associated with age of onset of cannabis use, such that earlier onset was related to worse performance (61–68, 118). In a longitudinal cohort study following participants up to age 38, adolescent-onset cannabis users were found to have greater decline in IQ than adult-onset users when correcting for pre-use educational scores (70). Another study found a bi-directional relationship between cannabis use and cognitive performance such that poorer short-term memory and working memory performance at age 13 (prior to initiation of cannabis use) was associated with earlier age of onset of cannabis use, and earlier onset and more frequent cannabis use during adolescence in turn was associated with decline in verbal IQ, executive function domains of trial, and error learning and reward processing by age 20 (69). It is worth noting that a meta-analysis of cross-sectional studies investigating the association between cognitive performance and cannabis use in adolescents and young adults reported only a modest overall effect which was no longer significant when considering studies of abstinent users, and also did not find any association with either age of cannabis user or age of onset of cannabis use (119). However, meta-analysis of existing studies that included participants with a wide range of age at recruitment and age of onset of cannabis use, as well as variability in the measurement of cannabis use, are inherently limited by heterogeneity in the data that was pooled to estimate the effect of interest, making any interpretation challenging.

### Studies Investigating Brain Structure Alterations Associated With Adolescent-onset Cannabis Use

A number of studies have employed magnetic resonance imaging (MRI) (please also see **Table 2** for a list of studies and summary results)to investigate alterations in brain structure associated with adolescent-onset of cannabis use and reported greater reduction in grey matter (73, 75, 83), hippocampal (81), and white matter (74, 77) volumes in adolescent-onset cannabis users compared to age-matched non-users. However, reduction in grey matter (82) and hippocampal (78) volumes have not always been consistently seen. Interestingly, earlier age of onset of cannabis use has also been associated with increase in white matter volume (83), orbitofrontal connectivity (120), and cortical thickness of the superior frontal gyrus (80). Deficits in white matter structural integrity have also been found in the prefrontal corpus callosum of adult cannabis users with onset of use in early adolescence compared to non-users (71). Further, frontotemporal structural connectivity has been found to be reduced in adolescent cannabis users compared to non-users (72), and similar alterations have been reported in the inferior longitudinal and inferior fronto-occipital fasciculi (white matter tracts connecting the occipital and temporal-occipital areas with the anterior-temporal regions (121) and the frontal lobe with temporal and occipital regions (122), respectively), with decreasing values at follow up as a function of the amount of cannabis used (76).

Consistent with this, a longitudinal study indicated that grey matter alterations found in the hippocampus, amygdala, and superior temporal gyrus of adolescent onset cannabis users do not change further following use during adulthood, suggesting that structural changes may be predominantly occurring in adolescent users (79), potentially as a result of altered pruning during adolescence.

### Studies Investigating Brain Functional Alterations Associated With Adolescent-Onset Cannabis Use

Functional magnetic resonance imaging (fMRI) (please also see **Table 3** for a list of studies and summary results)while performing cognitive activation tasks or at rest has been used to compare adolescent cannabis users to non-using adolescent control groups (84–90, 91, 96). Increased brain activity has been observed in cannabis-using adolescents compared to age-matched controls (i) during reward processing, in the middle frontal gyrus, parietal lobe, occipital gyrus, precuneus, caudate, cingulate, insula, and claustrum (84, 86); (ii) during inhibition, in the frontal and occipital gyri, parietal lobe, precuneus, and cingulate (85, 91); (iii) during memory performance, in the superior parietal lobe and cuneus (123), (iv) and during resting-state (96). The dorsolateral prefrontal cortex was found to have decreased activation in adolescent cannabis users compared to age-matched controls during memory performance (123). However, compared to non-using adolescent controls no altered activation was reported in adolescent cannabis users during reward (87) or inhibition (90) processing. Further, region of interest (ROI) analysis specific to task found hyperactivation in prefrontal regions during memory processing (124) and in the amygdala while fear processing (125) compared to controls. This hyperactivation may be as a result of increased effort to maintain task performance or reflect reduced cortical efficiency (117).

Using a whole-brain analysis approach, a meta-analysis of studies specifically investigating alteration in brain activation associated with cannabis use in adolescence, reported significantly greater activation in cannabis users compared to controls over a range of tasks in the right inferior parietal gyrus (extending to the superior parietal gyrus and angular gyrus) and right putamen (extending to the striatum and insula) (126). These regions are part of the salience and default mode networks (127, 128), potentially suggesting an impairment in the functioning of brain regions involved in the control and switching between states of carrying out a mental task and rest; necessary for the efficient allocation of attentional resources while performing mental tasks. Even following a period of abstinence long enough to allow cannabis metabolites to have left the body, these networks in adolescent cannabis users persisted to show altered activation (129). Interestingly, in contrast to the meta-analysis of adolescent cannabis user studies showing greater activation in cannabis users across a number of brain regions (126) a meta-analysis of adult only studies found brain functional alterations in both directions (i.e. hyperactivation and hypoactivation) (126). While not directly comparable, these differences may reflect a range of differences between study populations in the two meta-analyses, including differences in the extent of exposure to cannabis as well as other drugs. A subsequent study performed in adult cannabis users with a narrow range of age of onset of cannabis use during adolescence found evidence of inefficient medial temporal and midbrain function underlying slower verbal learning (130).

Despite being limited, functional imaging studies have also investigated whether early and late-onset cannabis users differed in their brain activity (92–94) while performing cognitive tasks. While performing memory tasks, early onset cannabis users had increased activation in the superior parietal lobe, the inferior and superior frontal and superior temporal gyri, insula, and precuneus (92), compared to late-onset users. While performing inhibitory processing tasks, early onset cannabis users had altered functioning where they activated different regions of the anterior cingulate cortex (ACC) compared to controls, while late onset users showed activation patterns that were similar to a control group of non-users (93, 94).

Other studies have investigated alteration in the functional connectivity between different regions of the brain in the context of cannabis use to complement and help better understand the differences in brain activation seen in cannabis users (126). A longitudinal study of resting state functional connectivity comparing adolescence cannabis users and non-users found decreased connectivity between the ACC and the dorsolateral and orbital frontal cortices in adolescent cannabis users across 18 months of cannabis use, while connectivity between the ACC and the superior frontal gyrus increased over time in healthy controls (97). Examination of resting state connectivity between the central executive network, default mode network and sensory networks in a cohort of adolescents found decreased connectivity in all networks as a function of longer duration of cannabis use (98). Similar alterations were seen in connectivity between the striatum and frontal–limbic circuit during a comparison of adolescent cannabis users with non-users, in addition to attenuation of the negatively correlated functional connectivity between the striatum and the fusiform gyrus, a region that serves a critical role in the recognition of significant visual features; it is important to note that some of these observations appeared to normalize after abstinence (95). Reduced interhemispheric connectivity in adolescent cannabis users compared to non-users has also been observed, associated with dependence levels (96).

### Preclinical Evidence of Neurobiological Alterations Associated With Adolescent Cannabis Use

Direct comparison of adolescent exposure with comparable exposure during adulthood is challenging to address in human studies. Preclinical studies have further advanced current understanding of the effects of cannabinoids in adolescence, with experimental designs allowing for systematic investigation and better control of potential confounding factors (please see **Table 4** for a list of studies and summary results). Behavioral studies of animals exposed to CB1 receptor agonists, including THC (99, 100, 102, 103), during adolescence compared to those exposed in adulthood found the former to have more significant cognitive impairments when compared to age-matched controls, while the latter had minimal impairment (101–104, 107, 108, 131). However, other studies did not find any difference in impairments comparing the adolescent exposed group to the adult exposed group (105) and some others did not find any deficit following chronic cannabinoid exposure in either the adolescent exposed group or the adult exposed group compared to controls (99, 100).

Histological investigations in preclinical studies suggest neurobiological changes in animals exposed to cannabinoids during adolescence. Adolescent THC-treated rats maintained increased expression of the CB1 gene compared to adult THC treated rats (103). Furthermore, adolescent animals had a reduction in compensatory downregulation of CB1 receptors following acute exposure to synthetic cannabinoids, compared to adults exposed animals (109). Protein expression in adolescence has also been found to be altered in prefrontal regions and the hippocampus (106, 132), possibly due to altered CB1 receptor-mediated regulation of downstream signaling proteins. Further, depression in GABA and glutamate receptors, leading to a disruption of the excitatory-inhibitory balance, has been observed in the hippocampus following adolescent cannabinoid exposure (110, 112) compared to animals treated with a vehicle, with an opposite effect on GABA receptors seen in adult treated rats (114). Similar findings in the prefrontal cortex (113, 115) and hyperactivity of mesocorticolimbic dopaminergic systems have been found in adolescent treated animals compared to animals treated with a vehicle (111).

## Discussion

As evident from our summary of current research, existing literature is generally consistent with the idea that cannabis use in adolescence is associated with neurocognitive changes. Meta-analytic evidence suggests greater functional activation in adolescent cannabis users compared to controls, whereas adult users show a combination of hyper- and hypo- activation in a number of brain regions. Functional connectivity between brain regions and within known functional circuits is altered in those with adolescent cannabis use and may underlie the observed differences in brain activation, perhaps from inefficient recruitment of regions required for task performance. Such disordered organization of brain circuitry during adolescence may underlie greater functional deficits in adolescent cannabis users than those starting use as adults. Preclinical evidence further supports the idea that the detrimental effects of cannabis use may be greater specifically as result of exposure during the developmental period.

Adolescence seems to be a period of vulnerability to change, with brain structural alterations associating with cannabis use (79). However, structural changes in cortical and subcortical regions do not show great consistency in terms of direction of change (133). Functional alteration in cannabis users, do show some consistency towards increased activation. However, evidence of the regional pattern of this change has been less consistent, possibly due to differences between studies in the cognitive tasks employed, although brain regions that are part of the large functional networks, such as the salience and default mode networks (127, 128) show more robust evidence for functional alteration in adolescent cannabis users (126, 134). Observed functional network alterations could stem from altered pruning consistent with evidence of alterations in white matter volume (74, 77), integrity (71) and connectivity (72, 76, 121, 122). Brain regions that are key components of these large scale networks that have been found to be affected in adolescent cannabis users, have also been shown to be acutely modulated by THC, the key psychoactive ingredient in cannabis, in experimental studies (135–137), indicating that the alterations noted in adolescent cannabis users are likely related to cannabis use as opposed to being linked to potential confounding factors that are challenging to control for in observational studies.

Early onset of cannabis use during adolescence has been associated with poorer performance in cognitive tasks. However, the extent to which these deficits persist following an adequate period of abstinence remains unclear (119, 129). Whether adaptive changes occur in terms of functioning, particularly after a period of abstinence, bringing cognitive performance of abstinent cannabis users up to the level of non-users despite residual differences in brain functional activation (129) remains to be tested.

Altered functioning of salience and reward networks as well as altered inhibitory control from early cannabis use has been suggested to increase susceptibility to risky behaviors, addiction, and dependence (138). Also, the acute psychoactive effects of cannabis have been found to differ between adults and adolescents (139, 140), with adolescents perceiving them less (139), which may potentially underlie early onset users often becoming more persistent users than those with a later age of onset (70). However, whether these persistent brain functional alterations underlie short-term or longer-term risks of mental, social, and behavioral disturbances (4, 141–145) in young people remains to be tested.

It is possible that altered cognitive function and alteration in the neural substrates underlying those cognitive processes as summarized above, may underlie poorer educational outcomes and increased school drop-out rate (68, 69, 145–151) in early-onset cannabis users. However, it is worth noting the lack of evidence in this regard. Further, other factors such as pre-existing characteristics (139, 149), lower IQ and poorer executive functioning predating cannabis use (152–155), and decreased time spent in schooling (156) may also increase the likelihood of poor educational outcomes independently. Poor school performance in turn has been associated with increased likelihood of development of a psychiatric disorder (157).

Another important question relates to whether evidence summarized above helps us understand potential mechanisms through which cannabis use during adolescence may increase the risk of onset for psychiatric disorders such as schizophrenia (3, 158, 159), depression (4, 160, 161) and substance use disorders (68, 162) in adolescent-onset users compared to those with onset of use later on in life. The precise neurobiological mechanisms underlying the association between cannabis use and increased risk of development of psychosis, especially what underlies greater risk in those with onset of use during adolescence remains unclear, though there are several potential candidate explanatory mechanisms. Impaired functioning following cannabis use in adolescence may alter the functioning of components of the salience network, such as the insula, involved in the switching between large scale brain networks (125) that is critical to the efficient allocation of cognitive resources. Involvement of components of this switching process in conjunction with altered functioning of cross-modal hubs such as the angular gyrus, involved in the integration and retrieval of multi-modal information (163) and in the allocation of attentional resources and attribution of meaning and salience in association with components of the salience network, may result in altered attribution of salience in the context of cannabis use (164), that is also thought to underlie symptoms of psychosis (165). Functional connectivity alterations between the default mode network and salience network as summarized here may underlie the psychopathology of psychosis (166) indicating a potential convergent mechanistic substrate. Alterations following adolescent-onset cannabis exposure in the efficiency of medial temporal cortex (130) also converge with medial temporal alterations seen in psychosis (167–169). Using cannabis during the critical developmental period of adolescence may also lead to alterations in two neurotransmitter systems, such as altered glutamate (170) and dopamine (171) signaling, both of which have been implicated in the etiology of psychosis (172). Investigation of adolescent-onset cannabis users suggest that glial function may also be altered following cannabis use (173) indicating another point of convergence between alterations noted in the context of cannabis use and in schizophrenia (174) suggesting a potential mechanistic explanation.

### Limitations of Current Evidence and Directions for Future Research

Research into the adverse effects of psychoactive drug use during adolescence has numerous challenges (175), particularly in terms of systematic quantification of extent of cannabis use (176), as frequency and duration of cannabis use along with participant age may play a key role in influencing any observed alterations in brain and behavior (5). Currently, there is a lack of adequately powered systematic studies with well-defined groups of early-onset and late-onset cannabis users matched for potential confounding factors such as levels of cannabis exposure, which may help begin to address the question whether cannabis use during adolescence is associated greater brain structural and functional alterations than later onset use. Similarly, three-way analysis of early-onset users, late-onset users, and age-matched controls would help identify areas of functional and structural alteration common to all cannabis users as well as alterations that are specifically associated with adolescent onset use. Neurochemical changes are also yet to be investigated in a way that adequately addresses difference between adolescent or adult-onset cannabis users and should be a consideration for future studies. In terms of its remit, this current review has been limited to the brain alterations associated with adolescent cannabis use, as indexed by evidence of structural, neurophysiological, and cognitive performance alterations. There is also a wider body of evidence regarding the association between adolescent cannabis use and an increased risk of development of emotional disturbances, psychosis or addiction, which were not reviewed here. Future efforts should also attempt to systematically summarize the longer-term mental health consequences of adolescent cannabis use and integrate them with biological evidence that may underpin them.

Important potential confounding factors that have not always been considered adequately but are worth consideration include abstinence (119, 129) and tolerance (177, 178) to the effects of cannabis. Future studies need to take these factors into consideration at the design stage. Another important consideration in this context relates to the issue of cause and effect relationship. In light of the cross-sectional design of the majority of studies, much of the currently available human evidence is unable to disentangle the nature of the relationship between adolescent cannabis use and neurocognitive alterations, i.e., whether adolescent cannabis use is a cause or consequence of these alterations or whether their association is linked to an underlying predisposition that increases the likelihood of both adolescent cannabis use and certain neurocognitive outcomes. More systematic longitudinal studies, which have been limited thus far, are necessary to start addressing some of these questions. Other approaches such as sibling/twin study designs (179, 180) or analytic approaches (4, 179, 181) as have been employed in the context of cannabis use and other adverse outcomes may also offer alternative methods of addressing these questions even in the absence of experimental data that constitute the gold standard evidence for establishing causal relationships. Future studies in large samples are also necessary to disentangle whether different sub-groups of adolescent cannabis users have differential vulnerability to the effects of cannabis, potentially mediated by genetic differences (182–185). Future studies also need to investigate whether the emergence of new cannabis preparations and increasing use of synthetic cannabis may have altered the usage patterns in adolescence and resulted in greater harm from adolescent use.

## Conclusions

While there is growing interest in the therapeutic potential of cannabis (186–188) and evidence of benefit only for certain cannabinoids such as cannabidiol for certain childhood epilepsies (189, 190), or potential for benefit for neurodevelopmental disorders such as schizophrenia (191–195) that typically have an onset in late adolescence and early adulthood, evidence summarized above indicate the need for caution. This is a particular concern as specific cannabinoids (such as cannabidiol) with therapeutic potential are often conflated with cannabis/medicinal cannabis in the public discourse leading to potential trivialization of possible harm from cannabis use in adolescent users and reinforcement of the narrative that cannabis use is a harmless recreational activity in young people. Collectively, despite the obvious limitations outlined above, current evidence indicates that adolescence is a sensitive period during which cannabis use may result in adverse neurocognitive effects that appear to show a level of permanency into adulthood.

## Author Contributions

SB and GB-H designed the study. GB-H performed the literature searches for the studies included and composed the initial draft, with substantial contributions for content and presentation from MC, VG, and SB.

## Funding

GB-H was supported by funding from the Society for the Study of Addictions for postdoctoral work with SB that included this review. SB is supported by grant from the National Institute of Health Research (NIHR) Efficacy and Mechanism Evaluation scheme (UK).

## Conflict of Interest

The authors declare that the research was conducted in the absence of any commercial or financial relationships that could be construed as a potential conflict of interest.
